# Triglycerides and Hypertension in a Korean Population: An Individual-Level Mendelian Randomization Analysis

**DOI:** 10.3390/nu18040633

**Published:** 2026-02-14

**Authors:** Ximei Huang, Minjoo Kim

**Affiliations:** Department of Food and Nutrition, College of Life Science and Nano Technology, Hannam University, Daejeon 34054, Republic of Korea

**Keywords:** hypertension, triglycerides, Mendelian randomization, genetic risk score, Korean population

## Abstract

**Background**: Although elevated triglyceride (TG) levels are consistently associated with hypertension in observational studies, whether TGs have a causal effect on hypertension remains uncertain, and evidence in East Asian populations is limited. **Methods**: We analyzed 2159 Korean adults (20–86 years) whose individual-level genetic and phenotypic data were obtained from a cross-sectional health check cohort. Candidate TG-associated genetic variants were identified using genome-wide association analysis and evaluated as instrumental variables (IVs). An individual-level, two-stage IV Mendelian randomization (MR) framework was applied to assess the potential effect of TGs on hypertension, alongside conventional observational analyses using logistic regression. **Results**: Three candidate TG-associated single-nucleotide polymorphisms (SNPs)—rs78115082 (*TRPC7*), rs117867615 (*TTLL1*), and rs34463296 (*LINC03019*)—were identified and combined to construct a weighted genetic risk score (GRS). Although all the instruments met the conventional strength criteria (F statistics > 10), they explained only a modest proportion of the variance in TG levels (partial R^2^, 0.008–0.020). Observational analyses showed a strong positive association between TG levels and hypertension (crude odds ratio [OR] = 2.12; 95% confidence interval [CI]: 1.76–2.54; adjusted OR = 1.43; 95% CI: 1.16–1.75). In contrast, MR estimates based on individual SNPs and the GRS were directionally positive but statistically nonsignificant, with wide CIs crossing the null, indicating limited precision. **Conclusions**: In this Korean cohort, observational analyses demonstrated a robust association between TG levels and hypertension, whereas individual-level MR provided inconclusive genetic evidence for a causal effect under the available instruments. The difference between the observational and genetic estimates is compatible with the finding that TG levels reflect broader cardiometabolic dysregulation rather than acting as an isolated causal determinant of hypertension. These findings underscore the need for larger studies with stronger, externally derived instruments to refine the causal inference in East Asian populations.

## 1. Introduction

**Figure 1:**
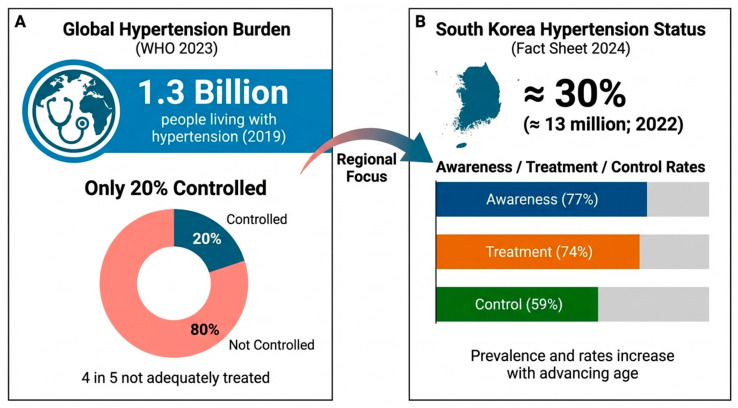
Global burden of hypertension and hypertension status in South Korea. (**Panel A**) summarizes the global burden of hypertension (≈1.3 billion adults with hypertension, 2019 estimate) and the low global control rate (≈20% controlled), as reported in the WHO global hypertension report (published in 2023) [2]. (**Panel B**) summarizes the estimated prevalence of hypertension in South Korea (≈30%, ≈13 million adults) and overall awareness, treatment, and control rates (77%/74%/59%) based on the Korea Hypertension Fact Sheet 2024 (data through 2022) [3].

Hypertension is one of the most important modifiable public health challenges worldwide and remains the leading preventable cause of premature mortality. It substantially increases the risk of cardiovascular events and target organ damage, contributing to a significant global disease burden [1]. The number of individuals living with hypertension has exceeded 1.3 billion globally, driven by population aging and adverse lifestyle changes, yet global blood pressure (BP) control rates remain below 20%, despite advances in diagnosis and treatment [2]. In the Republic of Korea, more than 13 million individuals were estimated to have hypertension in 2022, with the incidence increasing sharply with age [3]. Although age-standardized cardiovascular mortality has decreased in recent decades due to improved BP management, the persistently high prevalence of hypertension underscores the need for a deeper understanding of its underlying pathogenic mechanisms and modifiable determinants [3]. Contextual background on the key global and national indicators of the hypertension burden is summarized in Figure 1.

Hypertension is a complex, multifactorial condition that rarely arises through a single pathway. Approximately 85% of hypertensive patients present with at least one additional cardiovascular risk factor, and such clustering markedly amplifies the cardiovascular risk [4,5]. Among these factors, dyslipidemia—particularly elevated triglyceride (TG) levels—frequently coexists with hypertension and is associated with adverse cardiometabolic outcomes [6]. From a biological perspective, elevated TG levels are closely linked to insulin resistance (IR) and ectopic lipid handling, which can influence BP regulation through sympathetic nervous system activation, renal sodium retention, endothelial dysfunction, and vascular remodeling [7]. In addition, the cholesterol carried in TG-rich lipoproteins and remnant particles has been implicated in vascular inflammation and impaired endothelial signaling, suggesting that circulating TG levels may reflect broader cardiometabolic dysregulation rather than acting as a single, isolated driver of BP elevation [8]. Consistent with these mechanisms, numerous observational studies have reported positive associations between TG levels and the hypertension risk, even after conventional adjustment for confounders [9,10]. However, such associations remain susceptible to residual confounding and reverse causation, limiting their ability to establish causality or to disentangle whether TGs themselves is causally involved or primarily serve as a metabolic correlate of underlying dysfunction [11].

Mendelian randomization (MR) has emerged as a powerful epidemiological approach to strengthen causal inference using genetic variants as instrumental variables (IVs) for modifiable exposures. Because germline genetic variants are randomly allocated at conception, MR analyses are less vulnerable to confounding and reverse causation than conventional observational studies and thereby approximate aspects of a randomized controlled trial [12]. MR has been applied to investigate potential causal links between lipid traits, including TGs, and BP-related phenotypes, with some studies suggesting that genetically proxied TG levels may influence BP through intermediate pathways [8]. Nevertheless, much of the existing MR evidence for lipid traits and hypertension is derived from large, genome-wide association studies (GWASs) and relies on summary-level two-sample designs [13,14]. These approaches, while powerful, limited direct comparisons between observational and genetically informed estimates within the same population and cohort. Moreover, lipid-associated loci, allele frequencies, and effect sizes vary across ancestries, and several TG-related loci exhibit population-specific patterns, raising concerns about the transferability of European-derived instruments and causal estimates to East Asian populations [15,16]. Consequently, individual-level MR studies conducted within Korean or East Asian cohorts, which allow for a direct comparison between observational and genetic estimates under a shared confounding structure, remain limited but are of particular relevance.

Therefore, the primary objective of this study was to evaluate the potential causal relationship between TG levels and hypertension in a Korean population using an individual-level, two-stage IV MR framework. As a secondary, exploratory objective, we sought to identify TG-associated genetic variants within this cohort as candidate instruments for future external replication in East Asian populations. By contrasting MR-derived estimates with observational associations within the same cohort, this study aims to provide population-specific insights into the role of TGs in hypertension while appropriately delineating causal inference from metabolic correlations.

## 2. Materials and Methods

### 2.1. Study Population

This study was conducted using data from a previously established health check cohort at Ilsan Hospital, which is part of the National Health Insurance Service in Goyang, Republic of Korea. Participants underwent routine health examinations between January 2010 and March 2015 and provided individual-level phenotypic and genetic data. The study protocol was approved by the Institutional Review Board of Hannam University (project identification code: 2023-04-08-0405; approval date: 5 April 2024) and was conducted in accordance with the Declaration of Helsinki. All participants were informed of the study objectives and procedures and provided written informed consent before their enrollment.

Hypertension was defined according to the Korean Society of Hypertension guidelines as a systolic BP ≥ 140 mmHg, a diastolic BP ≥ 90 mmHg, or the current use of antihypertensive medication [3]. Medication use and medical history were ascertained using a standardized questionnaire; “regular users of medications” were defined as participants with current, ongoing use of prescription medications at the time of the health examination. Participants were excluded if they had recent diagnoses or a history of cardiovascular disease, liver disease, renal disease, pancreatitis, or cancer; were pregnant or lactating; regularly used prescription medications for conditions other than hypertension that could materially influence cardiometabolic biomarkers (e.g., lipid-lowering or glucose-lowering agents); or had missing or incomplete data. After applying these criteria, 2159 participants aged 20–86 years were included in the final analytical sample.

### 2.2. Clinical and Biochemical Assessments

The general anthropometric and sample collection procedures have been described elsewhere [17]. Briefly, body weight and height were measured, and the body mass index (BMI) was calculated in units of kilograms per square meter. Systolic and diastolic BP were measured in the seated position after a 20 min rest using an automatic BP monitor (FT-200S, Jawon Medical, Gyeongsan, Republic of Korea), and the average of two readings was used for analysis. Fasting blood and spot urine samples were collected after an overnight fast of at least 12 h.

Biochemical measurements included fasting glucose, insulin, hemoglobin A1c (HbA1c), free fatty acid, TG, total cholesterol, high-density lipoprotein cholesterol (HDL-C), low-density lipoprotein cholesterol (LDL-C), malondialdehyde (MDA), oxidized LDL (ox-LDL), and high-sensitivity C-reactive protein (hs-CRP) levels, which were measured using previously described methods. IR was estimated using the homeostasis model assessment (HOMA-IR) formula, as follows: (fasting glucose [mmol/L] × fasting insulin [μIU/mL])/22.5.

Urinary 8-epi-prostaglandin F_2α_ (8-epi-PGF_2α_) levels were quantified using an enzyme immunoassay with a Urinary Isoprostane ELISA kit (Oxford Biomedical Research, Rochester Hills, MI, USA). Serum apolipoprotein A-I (ApoA-I) and B (ApoB) levels were measured using turbidimetric immunoassays (Roche, Basel, Switzerland). The plasma ApoA-V concentration was determined using a Human apoA5 ELISA Kit (Cusabio, Wuhan, China). Serum interleukin (IL)-6, IL-1β, and tumor necrosis factor-alpha (TNF-α) levels were measured using commercially available immunoassay kits according to the manufacturer’s protocols.

### 2.3. Single-Nucleotide Polymorphism (SNP) Genotyping and Quality Control

Genotyping was performed using the Affymetrix Axiom^TM^ KORV1.1-96 Array with the Axiom^®^ 2.0 Reagent Kit (Affymetrix, Santa Clara, CA, USA). Approximately 200 ng of genomic DNA (gDNA) from each participant was amplified, fragmented, end-labeled with biotinylated nucleotides, hybridized to the array, and scanned using a GeneTitan^®^ MC Instrument (Affymetrix, Santa Clara, CA, USA). Genotypes were called using Genotyping Console^TM^ Software (Affymetrix, Santa Clara, CA, USA). Genotype data were generated using the Korean Chip (K-CHIP), developed by the Center for Genome Science at the Korea National Institute of Health and made available through the K-CHIP consortium.

Quality control excluded samples showing sex discrepancies, individual call rates < 90%, and SNPs with marker call rates < 95%, a minor allele frequency < 0.01, and Hardy–Weinberg equilibrium *p* < 0.001. Linkage disequilibrium (LD) pruning was applied only during instrument selection to obtain approximately independent candidate instruments (PLINK—indep-pairwise 250 50 0.5). After quality control, 394,222 SNPs and 2159 participants were included in the association analyses.

### 2.4. Genetic Instrument Selection and Genetic Risk Score (GRS) Construction

GWASs for TGs were conducted under an additive genetic model adjusted for age and sex using PLINK version 1.9 (https://www.cog-genomics.org/plink2 (accessed on 25 March 2025)). SNPs meeting the genome-wide suggestive significance threshold (*p* < 1 × 10^−5^) were considered candidate IVs. Although genome-wide significant variants (*p* < 5 × 10^−8^) are preferred, such variants were not available in this single cohort; therefore, a suggestive threshold commonly used in exploratory MR settings was adopted [18].

Recognizing the increased risk of false positives and the winner’s curse using this approach, candidate variants were treated as exploratory instruments and subjected to prespecified safeguards. These safeguards included an assessment of instrument strength (F statistic > 10), independence from major covariates (age, sex, and BMI), the absence of a direct association with hypertension, and negative-control regressions. Variants failing any prespecified IV assumption were excluded.

The final instrument set comprised three TG-associated SNPs. A TG-specific GRS was constructed by summing the number of risk alleles weighted by SNP-specific β-coefficients from first-stage linear regression models of TG levels adjusted for age, sex, and BMI. Because the SNP weights were derived within the same cohort, a prespecified K-fold cross-fitting procedure was additionally implemented to generate an out-of-fold weighted GRS as a sensitivity analysis.

### 2.5. MR Analysis

Because hypertension is a binary outcome, the primary individual-level MR analysis used a two-stage predictor substitution (2SPS) framework. In the first stage, linear regression models were used to estimate the associations between each IV and log-transformed TG levels (ln[TG]), adjusting for age, sex, and BMI. Instrument strength was assessed using the F statistic, with values >10 indicating adequate strength. In the second stage, the genetically predicted ln(TG) was entered into a logistic regression model to estimate its association with hypertension. Because a 2SPS framework with a nonlinear second-stage model for a binary outcome is an approximation and may be sensitive to model misspecification, a two-stage residual inclusion (2SRI) control function specification was prespecified as a robustness check by including first-stage residuals in the second-stage model. MR odds ratios (ORs) are interpreted per one-unit increase in genetically predicted ln(TG).

### 2.6. Statistical Analysis

**Figure 2:**
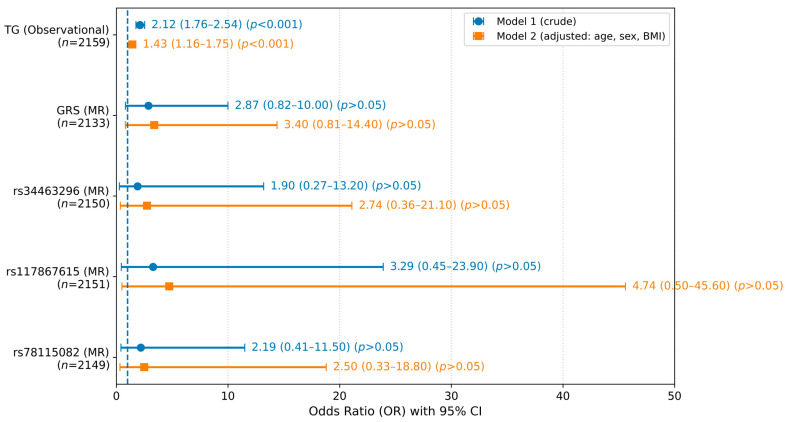
A forest plot comparing observational and individual-level Mendelian randomization (MR) estimates for triglycerides (TGs) and hypertension. Points denote ORs, and bars indicate 95% CIs for measured ln(TG) (observational) and genetically predicted ln(TG) using three SNPs and a weighted genetic risk score (GRS). The dashed line indicates OR = 1.0. Model 1 was the crude model; Model 2 was adjusted for age, sex, and BMI.

All statistical analyses were performed using SPSS Statistics version 26.0 (IBM Corp., Chicago, IL, USA) and RStudio-2025.05.0 (Posit PBC, Boston, MA, USA). Graphs used to visualize the data, including the forest plot (Figure 2), were generated using Python (version 3.10) with the matplotlib package. Continuous variables are presented as the means ± standard errors (SEs), and categorical variables are presented as frequencies and percentages. Variables with skewed distributions were logarithmically transformed to improve their normality. Differences in baseline characteristics between the normotensive and hypertensive groups were assessed using independent *t* tests for continuous variables and chi-square (χ^2^) tests for categorical variables. The UNIANOVA procedure was used to adjust for confounding factors. Logistic regression models were applied to estimate ORs and 95% confidence intervals (CIs) for the association with hypertension in both crude and adjusted models. Sensitivity analyses were prespecified to evaluate robustness and potential violations of MR assumptions. These analyses included (i) a 2SRI control function specification for the binary outcome, (ii) an expanded TG-increasing GRS including rs662799 (*APOA5*) to assess sensitivity to the instrument choice, (iii) an overidentified linear two-stage least squares (2SLS) model to enable Sargan and Wu–Hausman tests, (iv) covariate-adjusted negative control regressions of the IVs on other lipid traits and biomarkers, and (v) a K-fold cross-fitting procedure to mitigate within-cohort overfitting. Statistical significance was defined as a two-sided *p* < 0.05.

## 3. Results

### 3.1. Baseline Characteristics of Participants Stratified According to Their Hypertension Status

The baseline characteristics of normotensive (*n* = 1615) and hypertensive (*n* = 544) participants are presented in Table 1. Compared with normotensive individuals, participants with hypertension were more frequently male (52.0% vs. 36.2%) and older (54.4 vs. 48.3 years) and had a higher body weight, BMI, and waist circumference (all *p* < 0.001). As expected, both the systolic and diastolic BP levels were markedly higher in the hypertensive group, and these differences remained significant after adjusting for age, sex, and BMI (all *p*′ < 0.001).

With respect to metabolic and lipid parameters, fasting glucose, TG, and ApoA-V levels were higher in the hypertensive group and the differences remained significant after adjustment (*p*′ = 0.002, 0.004, and 0.014, respectively). HDL-C levels were lower in unadjusted comparisons, but the difference was attenuated after adjustment. In the adjusted comparisons, total cholesterol levels were higher (*p*′ = 0.017), whereas LDL-C (*p*′ < 0.001) and ApoA-I (*p*′ = 0.021) levels were lower.

With respect to oxidative stress and inflammatory markers, urinary 8-epi-PGF_2α_ levels remained significantly elevated after adjustment (*p*′ < 0.001). Associations with hs-CRP and IL-6 levels were attenuated after adjustment, whereas differences in TNF-α levels between groups persisted (*p*′ = 0.024).

Overall, several group differences persisted after adjustment, most notably higher fasting glucose, TG, ApoA-V, and urinary 8-epi-PGF_2α_ levels in the hypertensive group (Table 1).

### 3.2. Clinical and Biochemical Factors Associated with Hypertension

The associations between clinical/biochemical variables and hypertension are shown in Table 2. In unadjusted models, male sex, an older age, adiposity indices, glucose metabolism markers, TG-related variables, oxidative stress markers, and inflammatory markers were associated with higher odds of hypertension (all *p* < 0.05), whereas HDL-C levels were inversely correlated.

After adjusting for age, sex, and BMI, most associations were attenuated. Nevertheless, systolic and diastolic BP and fasting glucose, TG, ApoA-V, 8-epi-PGF_2α_, and TNF-α levels remained independently and positively associated with hypertension (all *p* < 0.05). Conversely, LDL-C and ApoB levels were inversely correlated, and ApoA-I was positively correlated according to the adjusted regression models (all *p* < 0.05). These adjusted directions for LDL-C/ApoB/ApoA-I levels are counter to conventional expectations; moreover, the direction of association for ApoA-I levels differs between adjusted group comparisons (Table 1) and adjusted regression models (Table 2), suggesting potential model-dependent effects. These findings are therefore interpreted cautiously and discussed further below.

In summary, after adjustment, TG levels and several correlates of cardiometabolic/oxidative stress remained positively associated with hypertension, whereas LDL-C and ApoB levels were inversely correlated according to the regression models (Table 2).

### 3.3. Selection of Genetic Instruments for MR

Based on the observational results, candidate IVs were explored for exposure traits associated with hypertension in both unadjusted and adjusted models, including fasting glucose, TG, ApoA-V, 8-epi-PGF_2α_, and TNF-α levels (Table 2). No genetic variants meeting prespecified IV screening criteria were identified for fasting glucose or TNF-α levels. For ApoA-V and 8-epi-PGF_2α_ levels, rs80339785 (*SCARA5*) and rs143222728 (*ABLIM3*) were initially identified but excluded because of insufficient instrument strength and/or a failure to meet prespecified validity checks (Table S1).

Among TG-associated variants, the well-established rs662799 (*APOA5*) variant was strongly associated with TG levels but was not included in the primary instrument set because its covariate-adjusted association with hypertension was directionally inconsistent with a TG-increasing instrument, raising concerns about potential violation of IV assumptions (Table S1). Consistent with our prespecified strategy, rs662799 was therefore evaluated only in an expanded GRS sensitivity analysis (Table S2). Ultimately, three TG-related SNPs—rs78115082 (*TRPC7*), rs117867615 (*TTLL1*), and rs34463296 (*LINC03019*)—met prespecified screening criteria and were selected for the primary MR analysis. None of these variants were significantly associated with age, sex, BMI, or hypertension (Table S3).

Overall, three TG-associated SNPs were retained for the primary MR models, whereas rs662799 (*APOA5*) was evaluated only in sensitivity analyses.

### 3.4. Associations Between Genetic Instruments and TG Levels

As shown in Table 3, all three SNPs and the weighted GRS were significantly associated with TG levels. In the crude models (Model 1), each SNP was positively associated with ln(TG) (β range, 0.071–0.164; all *p* < 0.001) and explained a small proportion of the variance in TG levels (R^2^ range, 0.008–0.011). The associations remained similar after adjusting for age, sex, and BMI (Model 2, all *p* < 0.001), with instrument-specific F statistics > 10 and modest partial R^2^ (0.008–0.010). The weighted GRS showed stronger associations with TG levels in both models (β = 0.610 in Model 1; β = 0.570 in Model 2), explaining a larger yet still modest proportion of variance (R^2^ = 0.019 in Model 1; partial R^2^ = 0.020 in Model 2) and yielding the greatest instrument strength (partial F ≈ 42).

Collectively, the selected instruments met the conventional strength criteria but explained only a modest proportion of the variance in TG levels, indicating that the precision of the downstream causal estimates was limited for this cohort (Table 3).

### 3.5. MR Estimates Compared with Observational Associations

Conventional observational analyses showed a strong association between measured ln(TG) and hypertension in both the crude and adjusted models (crude OR = 2.12; 95% CI: 1.76–2.54; adjusted OR = 1.43; 95% CI: 1.16–1.75; both *p* < 0.001) (Figure 2). The attenuation after adjustment was consistent with substantial confounding and/or shared cardiometabolic pathways captured by age, sex, and BMI.

In contrast, second-stage MR estimates based on genetically predicted ln(TG) were directionally positive (ORs > 1) but statistically nonsignificant in crude and adjusted models (all *p* > 0.05), with wide CIs crossing the null. In the adjusted model, the ORs ranged from 2.50 to 4.74 for the individual SNP instruments and were 3.40 for the GRS (Figure 2). As a robustness check for the binary outcome, 2SRI (control function) sensitivity analyses yielded directionally similar but imprecise estimates (Table S4).

Taken together, the results of the observational analyses clearly revealed a TG–hypertension association, whereas the MR analyses yielded directionally concordant but statistically inconclusive estimates with wide CIs, consistent with the limited precision in this cohort.

### 3.6. Sensitivity Analyses

In the expanded GRS analysis including rs662799 (*APOA5*), instrument strength increased as expected (partial F = 84.9; partial R^2^ = 0.038), yet the corresponding MR estimate remained directionally positive but statistically nonsignificant (adjusted OR = 2.46, 95% CI 0.87–6.97; *p* = 0.091) (Table S2).

For an overidentified sensitivity specification, we performed a linear 2SLS analysis (linear probability model) using the three TG instruments (Table S5). The results of the Sargan test were not significant (*p* = 0.848), and those of the Wu–Hausman test were not significant (*p* = 0.252) (Table S5). As negative control checks, we examined the associations between the TG instruments and other lipid traits/biomarkers (Table S6). Several nominal associations were observed without multiplicity correction; these results were presented for transparency and were not used for instrument selection or causal inference.

As a method to address potential optimism from within-cohort SNP weighting, K-fold cross-fitting was used to derive out-of-fold weights and a cross-fitted GRS, resulting in 2SPS and 2SRI estimates that were materially consistent with those of the primary analyses (Table S7).

Overall, across complementary sensitivity analyses, we did not observe evidence suggesting strong violations of the MR assumptions attributable to detectable horizontal pleiotropy; however, these diagnostics have limited power when the instruments explain only a modest proportion of the variance in TG levels.

## 4. Discussion

In this Korean health check cohort, conventional observational analyses revealed a positive association between TG levels and hypertension, which is consistent with the findings of previous studies across diverse populations [19,20]. However, such associations remain vulnerable to residual confounding and reverse causation [11]. In our data, the TG–hypertension association was attenuated substantially after adjustment for age, sex, and BMI, indicating that these correlates account for an important component of the observational relationship and motivate genetically informed analyses to better distinguish correlation from causation.

Across individual-level MR analyses, the estimated effects of genetically predicted TG levels on hypertension were directionally positive but statistically nonsignificant and imprecise. From a biological perspective, this pattern is compatible with TGs functioning as markers of broader cardiometabolic dysregulation, such as obesity, insulin resistance, and altered TG-rich lipoprotein metabolism, rather than acting as a single, isolated determinant of blood pressure regulation [19,21,22,23]. In this context, TG levels may allow us to track upstream metabolic and vascular pathways that coincide with an elevated BP without necessarily exerting a strong independent causal effect. Importantly, given the cross-sectional design and the limited precision of the MR estimates, this interpretation should be regarded as contextual and hypothesis-generating. Accordingly, while our findings do not provide strong genetic support for a causal role of TGs under the available instruments, they do not exclude the possibility of modest causal effects.

Methodologically, our MR results should be interpreted primarily through the lens of statistical precision. Although the selected instruments met conventional strength criteria (F statistics > 10) [24], they explained only a modest proportion of the variance in TG levels, which inherently limits power in individual-level MR—particularly with a binary outcome. Prior work and simulation studies indicate that very large sample sizes are often required to detect small-to-moderate causal effects when the instruments explain limited exposure variance [25,26]. Accordingly, the absence of statistical significance in the present MR analyses is best interpreted as inconclusive genetic evidence based on the available instruments rather than as definitive evidence against causality. Consistent with this interpretation, in the prespecified expanded GRS sensitivity analysis including rs662799 (*APOA5*), first-stage prediction improved, yet second-stage estimates remained uncertain with a wide CI.

Additional modeling considerations apply because hypertension is a binary outcome. Our primary specification used 2SPS with a logistic second stage; because nonlinear second-stage IV models represent an approximation and may be sensitive to misspecification [27], we prespecified complementary robustness analyses. These analyses included 2SRI, an overidentified linear probability 2SLS sensitivity model enabling Sargan and Wu–Hausman tests, negative-control regressions, and a cross-fitted GRS to mitigate optimism from within-cohort weighting. Across these prespecified checks, the estimates remained directionally concordant, and we did not observe definitive evidence suggesting major violations of the IV assumptions attributable to detectable horizontal pleiotropy, while recognizing that diagnostic power is limited when exposure prediction is modest.

Comparisons with large European-ancestry two-sample MR studies should be interpreted in light of differences in instrument construction, statistical power, and pleiotropy control. Large GWAS consortia typically provide numerous independent genome-significant lipid instruments, increasing explained variance and enabling broader pleiotropy-robust sensitivity analyses (e.g., MR–Egger and MR-PRESSO) [28,29]. In contrast, our one-sample MR analysis relied on a small set of cohort-specific candidate TG instruments, limiting both the precision and the applicability of multi-instrument pleiotropy-robust estimators. These differences underscore the need for replication in larger East Asian cohorts using stronger, independently derived TG instruments to refine the causal inference.

An additional contribution of this study is the identification of three TG-associated loci—rs78115082 near *TRPC7*, rs117867615 near *TTLL1*, and rs34463296 near *LINC03019*—in a Korean population. Because these variants were discovered and evaluated within the same cohort and lack external replication, they should be regarded as hypothesis-generating candidates rather than confirmed novel loci, and any functional interpretation remains preliminary. Limited functional annotation suggests that transient receptor potential cation channel subfamily C member 7 (*TRPC7*) is involved in calcium signaling pathways, which have been implicated in hepatic lipid metabolism, insulin signaling, and broader cardiometabolic regulation [30,31,32,33]. Tubulin tyrosine ligase-like 1 (*TTLL1*) encodes a protein involved in microtubule modification and cytoskeletal organization, processes linked to intracellular trafficking and metabolic regulation, including lipid handling [34]. The locus *LINC03019* resides in a long intergenic noncoding RNA region, which is consistent with accumulating evidence that long noncoding RNAs play regulatory roles in lipid and lipoprotein metabolism [35,36,37]. Collectively, these loci extend the descriptive landscape of TG-associated genetic variation in a non-European population and provide candidates for future external replication and functional follow-up.

Despite identifying plausible genetic IVs for TG, we were unable to establish instruments meeting prespecified screening criteria for several other metabolic and inflammatory markers associated with hypertension in observational analyses, including fasting glucose, ApoA-V, 8-epi-PGF_2α_, and TNF-α levels. This result reflects the multifactorial nature of hypertension and the challenge of a causal inference in correlated metabolic systems, where multiple biomarkers cluster together but lack sufficiently strong and specific genetic instruments within a single cohort. Consequently, although these traits showed cross-sectional associations with hypertension, our analyses do not permit causal isolation of their individual effects, underscoring the need for larger samples, stronger instruments, and/or longitudinal designs.

Several associations with lipid-related molecules were observed after the model with multivariable adjustment deviated from conventional expectations. LDL-C and ApoB levels were inversely associated with hypertension, whereas ApoA-I levels were positively associated. These directions contradict established evidence regarding the role of ApoB-containing lipoproteins and cumulative LDL exposure in atherosclerotic cardiovascular disease [38,39] and therefore warrant a cautious interpretation. Plausible noncausal explanations include reverse causation and selection effects in cross-sectional data, whereby single-timepoint lipid measurements may reflect behavioral or metabolic changes following a recognition of the cardiometabolic risk, as well as potential distortion from exclusion of regular lipid- or glucose-lowering medication users [40]. In addition, multivariable adjustment may induce conditioning (collider or overadjustment) bias, which can produce sign reversals in regression coefficients [41,42]. Accordingly, these findings are interpreted as model-dependent and hypothesis-generating, and further clarification will require longitudinal analyses and more detailed lipoprotein phenotyping.

This study had several strengths, including the use of individual-level data that enabled direct comparisons between observational and IV-based estimates within the same cohort. In addition, instrument selection and MR analyses followed a prespecified workflow incorporating multiple safeguards and sensitivity analyses, including alternative specifications and cross-fitting to mitigate optimism from within-cohort weighting. Nevertheless, important limitations should be acknowledged. The analysis was restricted to a Korean population and a cross-sectional design, limiting generalizability and temporal inference. Most importantly, the modest proportion of TG variance explained by the genetic instruments constrained MR precision such that small-to-moderate causal effects could not be excluded. Residual confounding by unmeasured lifestyle or environmental factors may persist in observational models, and external replication using stronger, independently derived instruments will be needed to refine causal conclusions.

## 5. Conclusions

In this Korean cohort, TG levels were strongly associated with hypertension in observational analyses, whereas individual-level MR analysis yielded directionally positive but statistically nonsignificant and imprecise causal estimates. Given the modest variance explained by the genetic instruments and the resulting wide CIs, the genetic evidence for a causal effect of TG on hypertension remains inconclusive, and modest causal effects cannot be excluded. The discrepancy between observational and genetic estimates is compatible with the use of TG, reflecting broader cardiometabolic dysregulation rather than acting as an isolated causal determinant of hypertension within this cross-sectional setting. Larger studies in East Asian populations that use stronger and externally derived genetic instruments are needed to improve precision and refine causal inference.

## Figures and Tables

**Table 1:** Comparison of baseline characteristics between normotensive and hypertensive individuals.

Variables	Normal (*n* = 1615)	Hypertension (*n* = 544)	*p*	*p*′
Male/Female *n*, (%)	585 (36.2)/1030 (63.8)	283 (52.0)/261 (48.0)	<0.001	—
Age (years)	48.3 ± 0.27	54.4 ± 0.50	<0.001	—
Weight (kg)	63.0 ± 0.25	68.0 ± 0.51	<0.001	0.083
BMI (kg/m^2^)	23.7 ± 0.07	25.4 ± 0.14	<0.001	—
Waist (cm)	83.5 ± 0.19	87.9 ± 0.37	<0.001	0.639
Systolic BP (mmHg)	116.4 ± 0.29	138.5 ± 0.66	<0.001	<0.001
Diastolic BP (mmHg)	72.7 ± 0.22	87.4 ± 0.46	<0.001	<0.001
Glucose (mg/dL) *^†^*	95.6 ± 0.51	103.8 ± 1.11	<0.001	0.002
Insulin (μIU/mL) *^†^*	9.09 ± 0.12	9.83 ± 0.25	0.026	0.600
HOMA-IR *^†^*	2.15 ± 0.03	2.55 ± 0.09	<0.001	0.143
HbA1c (%) *^†^*	6.10 ± 0.04	6.24 ± 0.06	0.029	0.865
Free fatty acids (μEq/L) *^†^*	552.4 ± 6.35	579.3 ± 11.6	<0.001	0.170
TG (mg/dL) *^†^*	119.6 ± 1.84	148.6 ± 3.75	<0.001	0.004
Total cholesterol (mg/dL) *^†^*	198.0 ± 0.90	198.3 ± 1.54	0.935	0.017
HDL-cholesterol (mg/dL) *^†^*	53.9 ± 0.34	50.4 ± 0.55	<0.001	0.407
LDL-cholesterol (mg/dL) *^†^*	121.0 ± 0.82	119.0 ± 1.42	0.194	<0.001
ApoA-I (mg/dL) *^†^*	156.1 ± 0.91	155.4 ± 1.37	0.735	0.021
ApoB (mg/dL) *^†^*	104.4 ± 0.94	103.9 ± 1.36	0.994	0.058
ApoA-V (ng/mL) *^†^*	284.8 ± 9.17	319.7 ± 15.5	0.012	0.014
MDA (nmol/mL) *^†^*	8.88 ± 0.08	9.72 ± 0.26	0.069	0.382
Oxidized LDL (U/L) *^†^*	46.1 ± 0.53	48.3 ± 0.97	0.017	0.929
8-epi-PGF_2α_ (pg/mg creatinine) *^†^*	1437.6 ± 18.9	1768.5 ± 53.6	<0.001	<0.001
hs-CRP (mg/L) *^†^*	1.22 ± 0.07	1.48 ± 0.12	0.001	0.629
TNF-α (pg/mL) *^†^*	10.9 ± 1.01	10.7 ± 1.03	0.006	0.024
IL-1β (pg/mL) *^†^*	0.93 ± 0.10	0.78 ± 0.05	0.163	0.473
IL-6 (pg/mL) *^†^*	3.62 ± 0.11	3.98 ± 0.22	0.011	0.106

The data are presented as frequencies (%) and means ± standard errors (SEs). Variables marked with *^†^* were ln-transformed for analyses; descriptive values are shown on the original scale. *p* values were obtained using independent *t* tests (continuous variables) or chi-square tests (categorical variables). *p*′ values were adjusted for age, sex, and BMI. 8-epi-PGF_2α_, 8-epi-prostaglandin F_2α_; Apo, apolipoprotein; BMI, body mass index; HbA1c, hemoglobin A1c; HDL, high-density lipoprotein; HOMA-IR, homeostasis model assessment–insulin resistance; hs-CRP, high-sensitivity C-reactive protein; IL, interleukin; LDL, low-density lipoprotein; MDA, malondialdehyde; TG, triglyceride; TNF-α, tumor necrosis factor-alpha.

**Table 2:** Associations of clinical and biochemical variables with hypertension.

Variables	Unadjusted			Adjusted		
	OR	95% CI	*p*	OR	95% CI	*p*
Sex (Female vs. Male)	0.52	0.43–0.64	<0.001	—	—	—
Age (years)	1.05	1.04–1.06	<0.001	—	—	—
Weight (kg)	1.05	1.03–1.05	<0.001	1.02	0.99–1.05	0.092
BMI (kg/m^2^)	1.19	1.15–1.23	<0.001	—	—	—
Waist (cm)	1.06	1.06–1.08	<0.001	1.00	0.98–1.02	0.896
Systolic BP (mmHg)	1.15	1.14–1.17	<0.001	1.14	1.13–1.16	<0.001
Diastolic BP (mmHg)	1.19	1.17–1.21	<0.001	1.19	1.17–1.21	<0.001
Glucose (mg/dL) *^†^*	7.27	4.40–12.0	<0.001	2.20	1.28–3.80	0.005
Insulin (μIU/mL) *^†^*	1.31	1.05–1.63	0.018	1.04	0.80–1.34	0.792
HOMA-IR *^†^*	1.64	1.34–2.01	<0.001	1.15	0.91–1.45	0.245
HbA1c (%) *^†^*	4.95	1.16–21.2	0.031	1.26	0.25–6.43	0.781
Free fatty acids (μEq/L) *^†^*	1.23	0.99–1.53	0.060	1.18	0.92–1.50	0.194
TG (mg/dL) *^†^*	2.12	1.76–2.54	<0.001	1.43	1.16–1.75	<0.001
Total cholesterol (mg/dL) *^†^*	1.02	0.60–1.73	0.935	0.61	0.34–1.09	0.094
HDL-cholesterol (mg/dL) *^†^*	0.34	0.23–0.51	<0.001	0.79	0.51–1.23	0.304
LDL-cholesterol (mg/dL) *^†^*	0.80	0.57–1.12	0.194	0.54	0.37–0.78	0.001
ApoA-I (mg/dL) *^†^*	0.90	0.48–1.69	0.896	2.18	1.09–4.38	0.028
ApoB (mg/dL) *^†^*	0.99	0.67–1.49	0.994	0.62	0.40–0.97	0.036
ApoA-V (ng/mL) *^†^*	1.23	1.05–1.45	0.012	1.26	1.05–1.51	0.012
MDA (nmol/mL) *^†^*	1.43	1.04–1.96	0.028	0.86	0.61–1.22	0.398
Oxidized LDL (U/L) *^†^*	1.37	1.06–1.77	0.017	1.08	0.81–1.44	0.601
8-epi-PGF_2α_ (pg/mg creatinine) *^†^*	1.86	1.49–2.32	<0.001	1.90	1.50–2.41	<0.001
hs-CRP (mg/L) *^†^*	1.16	1.06–1.26	0.001	0.99	0.90–1.09	0.853
TNF-α (pg/mL) *^†^*	1.19	1.04–1.36	0.011	1.19	1.03–1.37	0.018
IL-1β (pg/mL) *^†^*	0.89	0.76–1.05	0.163	0.94	0.79–1.12	0.473
IL-6 (pg/mL) *^†^*	1.20	1.04–1.38	0.011	1.13	0.97–1.32	0.105

The values are presented as odds ratios (ORs) and 95% confidence intervals (CIs) obtained from logistic regression analyses. Variables marked with *^†^* were entered as ln-transformed values; thus, ORs represent the change in hypertension odds per one-unit increase in ln(variable). Unadjusted ORs were obtained using univariable models. Adjusted ORs were obtained from multivariable models that controlled for age, sex, and BMI. For sex, males were used as the reference category (females vs. males). 8-epi-PGF_2α_, 8-epi-prostaglandin F_2α_; Apo, apolipoprotein; BMI, body mass index; CI, confidence interval; HbA1c, hemoglobin A1c; HDL, high-density lipoprotein; HOMA-IR, homeostasis model assessment–insulin resistance; hs-CRP, high-sensitivity C-reactive protein; IL, interleukin; LDL, low-density lipoprotein; MDA, malondialdehyde; TG, triglyceride; TNF-α, tumor necrosis factor-alpha.

**Table 3:** Associations between selected SNPs/GRS and triglyceride levels.

SNP	N	Chr *^a^*	Gene *^a^*	Ref/Alt *^a^*	Model 1				Model 2			
					Beta (SE)	F	R^2^	*p*	Beta (SE)	F	R^2^	*p*
rs78115082	2149	*5*	*TRPC7*	G/T	0.164 (0.034)	23.4	0.011	<0.001	0.145 (0.032)	21.2	0.010	<0.001
rs117867615	2151	*22*	*TTLL1*	C/T	0.073 (0.018)	17.0	0.008	<0.001	0.069 (0.016)	17.9	0.008	<0.001
rs34463296	2150	*8*	*LINC03019*	C/T	0.071 (0.017)	18.0	0.008	<0.001	0.072 (0.016)	21.7	0.010	<0.001
GRS	2133	*—*	—	—	0.610 (0.094)	42.2	0.019	<0.001	0.570 (0.087)	42.7	0.020	<0.001

*^a^* Chromosomal location and gene annotation were obtained from the original reports. Beta coefficients (SE), F statistics, R^2^, and *p* values were obtained from linear regression analyses. TG levels were logarithmically transformed prior to the analysis. For the GRS, SNP-specific weights were the first-stage regression coefficients (β) estimated in this cohort (same analytic dataset). Model 1 is the unadjusted model (ln[TG] ~ instrument), and the reported R^2^ is the multiple R-square value of this model. Model 2 is adjusted for age, sex, and BMI (ln[TG] ~ instrument + age + sex + BMI); the reported R^2^ is the instrument-specific partial R^2^ value (variance explained by the instrument conditional on covariates), approximated as partial R^2^ = t^2^/(t^2^ + df), where t is the first-stage t statistic for the instrument and df is the residual degrees of freedom. For a single instrument parameter, the corresponding instrument-specific partial F statistic equals t^2^. BMI, body mass index; Chr, chromosome; GRS, genetic risk score; N, sample size; Ref/Alt, reference/alternative allele; SE, standard error; SNP, single-nucleotide polymorphism; TG, triglyceride.

## Data Availability

The data presented in this study are available on request from the corresponding author.

## Supplementary Materials

The following supporting information can be downloaded at https://www.mdpi.com/article/10.3390/nu18040633/s1: Table S1. Instrumental variable estimates for candidate genetic instruments of exposure traits; Table S2. Sensitivity analysis including rs662799 (APOA5): Instrument strength and individual-level MR estimates for TG levels and hypertension; Table S3. Associations of triglyceride-related genetic instruments with potential confounders and hypertension; Table S4. Two-stage residual inclusion (2SRI) logistic sensitivity analysis of hypertension; Table S5. Sensitivity analysis (linear probability model for hypertension) and overidentification diagnostics; Table S6. Associations of TG instruments with other lipid traits and biomarkers (negative-control checks); Table S7. Cross-fitted weighted GRS sensitivity analysis: Primary 2SPS and complementary 2SRI individual-level MR estimates for TGs and hypertension.

## Abbreviations

2SLS	Two-stage least squares
2SPS	Two-stage predictor substitution
2SRI	Two-stage residual inclusion
8-epi-PGF_2α_	8-epi-prostaglandin F_2α_
Apo	Apolipoprotein
BMI	Body mass index
BP	Blood pressure
CI	Confidence interval
GRS	Genetic risk score
GWAS	Genome-wide association study
HbA1c	Hemoglobin A1c
HDL-C	High-density lipoprotein cholesterol
HOMA	Homeostasis model assessment
hs-CRP	High-sensitivity C-reactive protein
IL	Interleukin
IR	Insulin resistance
IV	Instrumental variable
K-CHIP	Korean chip
LDL	Low-density lipoprotein
MDA	Malondialdehyde
MR	Mendelian randomization
OR	Odds ratio
ox-LDL	Oxidized low-density lipoprotein
SNP	Single-nucleotide polymorphism
TG	Triglyceride
TNF-α	Tumor necrosis factor-alpha
