# Supplementary material for: Triglycerides and Hypertension in a Korean Population: An Individual-Level Mendelian Randomization Analysis
Source: Nutrients. 2026 Feb 14;18(4):633. doi: 10.3390/nu18040633 (PMC12942852; doi:10.3390/nu18040633)
Supplement: Supplementary file 1 [file nutrients-18-00633-s001.zip › nutrients-4110311-supplementary.pdf]

Table S1. Instrumental Variable Estimates for Candidate Genetic Instruments of Exposure Traits

SNP	N	Associated Trait	Chr[a]	Gene[a]	Ref/Alt[a]	Stage 1					Stage 2		
						Beta	SE	F	R^2	*P*	Beta	SE	*P*
rs662799	2,153	TG	11	*APOA5*	G/A	0.187	0.017	127.2	0.056	<0.001	-0.117	0.444	0.793
rs80339785	1,560	ApoA-V	8	*SCARA5*	C/T	0.147	0.031	21.7	0.014	<0.001	-0.657	0.789	0.405
rs143222728	1,901	8-epi-$PGF_{2\alpha}$	5	*ABLIM3*	G/A	0.295	0.066	20.0	0.010	<0.001	1.472	1.135	0.195

[a]Information from the original report is shown. . Stage 1 (SNP → exposure) estimates were obtained from linear regression models adjusted for age, sex, and BMI, with exposure traits analyzed on the natural log scale (ln) when indicated. The instrument-specific F-statistic for the SNP term was calculated as t^2 from the first-stage regression, and the instrument-specific partial R^2 was approximated as $t^2/(t^2 + df)$, where df is the residual degrees of freedom. Stage 2 estimates were obtained from logistic regression models of hypertension on genetically predicted exposure values (predicted from Stage 1) adjusted for age, sex, and BMI; Beta coefficients represented log odds. These candidate instruments were not carried forward to the main Mendelian randomization (MR) analyses based on prespecified prioritization and/or lack of robust evidence in the two-stage instrumental variable analyses.

8-epi-$PGF_{2\alpha}$, 8-epi-prostaglandin $F_{2\alpha}$; ApoA-V, apolipoprotein A-V; Chr, chromosome; N, sample size; Ref/Alt, reference/alternative allele; SE, standard error; SNP, single nucleotide polymorphism; TG, triglycerides.

Table S2. Sensitivity Analysis Including rs662799 (*APOA5*): Instrument Strength and Individual-Level MR Estimates for TG and Hypertension.

Panel A. First-stage associations of the expanded wGRS with TG and instrument strength metrics

Instrument	N	Model 1				Model 2			
		Beta (SE)	F	R^2	*P*	Beta (SE)	F	R^2	*P*
Expanded GRS	2,128	0.509 (0.058)	76.7	0.035	<0.001	0.496 (0.054)	84.9	0.038	<0.001

Panel B. Second-stage individual-level MR estimates for hypertension using genetically predicted TG from the expanded wGRS

Model	β (SE)	OR	95% CI	*P*
Model 1 (unadjusted)	0.744 (0.478)	2.105	0.825–5.373	0.119
Model 2 (adjusted for age, sex, BMI)	0.900 (0.532)	2.460	0.868–6.974	0.090

The expanded GRS was constructed from four TG-associated variants (rs662799, rs78115082, rs117867615, and rs34463296) in the complete-case analytic set (N = 2,128). SNP-specific weights were obtained as the regression coefficients for each SNP from first-stage linear regression models of ln(TG) adjusted for age, sex, and BMI within the same analytic set; the GRS was computed as the sum of weighted risk-allele dosages.

In Panel A, Model 1 represents the unadjusted first-stage association between GRS and ln(TG), and Model 2 represents the association adjusted for age, sex, and BMI. R^2 in Model 1 corresponds to the variance explained in the unadjusted model. Because overall model R^2 in Model 2 reflects both the instrument and covariates, instrument-specific variance explained is reported as partial R^2, approximated from the t-statistic for the instrument as partial $R^2 = t^2/(t^2 + df)$, where df is the residual degrees of freedom from the first-stage model. For a single instrument term, the instrument-specific partial F-statistic equals t^2. In Panel B, second-stage estimates were obtained from logistic regression models of hypertension on genetically predicted ln(TG) (predicted from Panel A), with covariate adjustment as indicated. β denotes log-odds and OR = exp(β).

BMI, body mass index; CI, confidence interval; GRS, genetic risk score; MR, Mendelian randomization; N, sample size; OR, odds ratios; SE, standard error; TG, triglycerides.

Table S3. Association of Triglyceride-Related Genetic Instruments with Potential Confounders and Hypertension

Variable	Beta	SE	*P*
**rs78115082 (*n*=2,149)**			
Age	-0.754	0.707	0.286
Sex	-0.047	0.030	0.123
BMI	0.315	0.190	0.098
Hypertension	0.128	0.139	0.356
**rs117867615 (*n*=2,151)**			
Age	-0.396	0.368	0.281
Sex	0.005	0.016	0.770
BMI	0.127	0.098	0.195
Hypertension	0.086	0.073	0.239
**rs34463296 (*n*=2,150)**			
Age	-0.076	0.349	0.828
Sex	0.006	0.015	0.693
BMI	0.002	0.094	0.981
Hypertension	0.046	0.070	0.517
**GRS (*n*=2,133)**			
Age	-1.401	1.970	0.477
Sex	-0.060	0.085	0.479
BMI	0.759	0.528	0.151
Hypertension	0.643	0.389	0.099

Associations between each instrumental SNP/GRS and potential confounders (age, sex, and BMI) were tested using linear regression, and the associations with hypertension were tested using logistic regression. For hypertension, the beta coefficients represented log-odds (OR = exp[Beta]).
BMI, body mass index; GRS, genetic risk score; SE, standard error; SNP, single nucleotide polymorphism.

Table S4. Two-Stage Residual Inclusion (2SRI) Logistic Sensitivity Analysis for Hypertension.

Instrument	N	Stage 1		Stage 2			
		partial R^2	partial F	OR	95% CI	*P*	*P* (residual term)
rs78115082	2,149	0.010	21.2	2.50	0.35–18.1	0.363	0.574
rs117867615	2,151	0.008	17.9	4.75	0.48–46.5	0.181	0.302
rs34463296	2,150	0.010	21.7	2.61	0.33–20.6	0.363	0.570
wGRS	2,133	0.020	45.7	3.37	0.82–13.8	0.092	0.236

Two-stage residual inclusion (2SRI) was implemented as a sensitivity analysis appropriate for a binary outcome. In Stage 1, ln(TG) was regressed on each instrument and covariates (age, sex, BMI) using linear regression to obtain predicted values and residuals. In Stage 2, hypertension was modeled using logistic regression as: hypertension ~ ln(TG) + first-stage residual + age + sex + BMI, where residual = ln(TG) − predicted ln(TG). ORs and 95% CIs are reported as exp(β) for the ln(TG) coefficient. Stage-2 standard errors were computed using heteroskedasticity-consistent (HC1) estimators. Sample sizes vary across SNP instruments due to instrument-specific genotype availability; the GRS analysis required non-missing genotypes for all three SNPs.

BMI, body mass index; CI, confidence interval; GRS, genetic risk score; N, sample size; OR, odds ratios; TG, triglycerides.

Table S5. Sensitivity Analysis (Linear Probability Model for Hypertension) and Overidentification Diagnostics.

**Panel A. Linear 2SLS (LPM for hypertension) estimates**			
**Exposure**	**Estimate (β)**	**SE**	***P***
TG	0.171	0.107	0.110
Age	0.008	0.001	<0.001
Sex	-0.087	0.024	<0.001
BMI	0.020	0.007	0.002
**Panel B. Diagnostic tests**			
**Test**	**Statistic**	**df1 / df2**	***P***
Weak instruments (first-stage F test)	19.262	3 / 2126	<0.001
Wu–Hausman (endogeneity test)	1.315	1 / 2127	0.252
Sargan (overidentification test)	0.330	2 / NA	0.848

This sensitivity analysis used a linear probability model (LPM) for hypertension within a linear 2SLS framework to enable overidentification diagnostics. Instruments were rs78115082, rs117867615, and rs34463296, with age, sex, and BMI included as exogenous covariates in both stages. Diagnostic tests were obtained from the ivreg model output. Robust standard errors were computed using heteroskedasticity-consistent covariance estimates (HC1). A non-significant Sargan overidentification test was interpreted as no evidence of detectable violations of the exclusion restriction under the overidentified linear specification (i.e., no detectable instrument-inconsistent residual correlation attributable to horizontal pleiotropy); however, the test has limited power when instrument effects are small.

2SLS, two-stage least squares; BMI, body mass index; df, degrees of freedom (df1/df2 denote numerator/denominator degrees of freedom for the reported tests); SE, standard error; TG, triglycerides.

Table S6. Associations of TG Instruments with Other Lipid Traits and Biomarkers (Negative-Control Checks).

Biomarker	N	Instrument	Beta	SE	*P*	partial R²
Glucose (mg/dL)[†]	2,132	rs78115082	0.008	0.011	0.458	<0.001
		rs117867615	0.000	0.006	0.952	<0.001
		rs34463296	0.004	0.005	0.446	<0.001
		wGRS	0.044	0.031	0.151	0.001
LDL-cholesterol (mg/dL)[†]	2,106	rs78115082	0.014	0.018	0.421	<0.001
		rs117867615	-0.014	0.009	0.118	0.001
		rs34463296	-0.004	0.009	0.636	<0.001
		wGRS	-0.013	0.048	0.794	<0.001
ApoA-I (mg/dL)[†]	1,353	rs78115082	0.022	0.013	0.100	0.002
		rs117867615	-0.009	0.007	0.227	0.001
		rs34463296	-0.002	0.007	0.772	<0.001
		wGRS	-0.011	0.038	0.776	<0.001
ApoB (mg/dL)[†]	1,353	rs78115082	0.044	0.021	0.038	0.003
		rs117867615	0.014	0.011	0.229	0.001
		rs34463296	0.011	0.011	0.321	0.001
		wGRS	0.134	0.060	0.026	0.004
ApoA-V (ng/mL)[†]	1,546	rs78115082	-0.083	0.054	0.126	0.002
		rs117867615	-0.035	0.028	0.209	0.001
		rs34463296	-0.054	0.027	0.042	0.003
		wGRS	-0.431	0.151	0.004	0.005
8-epi-PGF$_{2\alpha}$ (pg/mg creatinine)[†]	1,896	rs78115082	-0.034	0.032	0.294	0.001
		rs117867615	0.017	0.017	0.298	0.001
		rs34463296	-0.013	0.016	0.399	<0.001
		wGRS	0.045	0.088	0.612	<0.001
TNF-α (pg/mL)[†]	1,633	rs78115082	0.047	0.063	0.460	<0.001
		rs117867615	0.071	0.033	0.031	0.003
		rs34463296	0.022	0.031	0.483	<0.001
		wGRS	0.223	0.174	0.200	0.001

Variables marked with [†] were entered as ln-transformed values. Each biomarker was regressed on each instrument separately using linear regression adjusted for age, sex, and BMI. β represents the adjusted association per effect allele (for individual SNPs) or per one-unit increase in GRS. Instrument-specific variance explained was summarized as partial R^2, derived from the instrument t-statistic as partial $R^2 = t^2/(t^2 + df)$, where df is the residual degrees of freedom from the corresponding regression model. Values are rounded to three decimals; therefore, absolute β values < 0.0005 are displayed as 0.000. These analyses were conducted as negative-control checks for potential horizontal pleiotropy and were not used for instrument selection.

8-epi-PGF$_{2\alpha}$, 8-epi-prostaglandin F$_{2\alpha}$; Apo, apolipoprotein; BMI, body mass index; df, residual degrees of freedom; GRS, genetic risk score; LDL, low-density lipoprotein; N, sample size; SE, standard error; SNP, single-nucleotide polymorphism; TG, triglycerides; TNF-α, tumor necrosis factor-alpha.

Table S7. Cross-fitted weighted GRS sensitivity analysis: primary 2SPS and complementary 2SRI individual-level MR estimates for TGs and hypertension.

Instrument	Stage 1		Stage 2 (2SPS)			Stage 2 (2SRI)			
	R^2	F	OR	95% CI	*P*	OR	95% CI	*P*	Residual *P*
Cross-fitted GRS	0.023	49.9	3.55	0.93–13.58	0.064	3.51	0.91–13.50	0.068	0.189

The cross-fitted weighted GRS was constructed from three TG-associated variants (rs78115082, rs117867615, rs34463296) using K-fold cross-fitting (K = 5). In each fold, SNP-specific weights were estimated in the training subset from first-stage linear regression of ln(TG) adjusted for age, sex, and BMI, and then applied to the held-out subset to compute an out-of-fold wGRS; out-of-fold scores were concatenated to form the cross-fitted GRS in the full analytic set (complete cases, N = 2,133).

Stage 1 was fit in the full analytic set as: ln(TG) ~ cross-fitted wGRS + age + sex + BMI. Instrument strength is summarized using the instrument-specific partial R^2, approximated as partial $R^2 = t^2/(t^2 + df)$, and the corresponding partial F-statistic ($= t^2$), where t is the first-stage t-statistic for the wGRS term and df is the residual degrees of freedom.

Stage 2 (primary 2SPS logistic MR) was fit as: hypertension ~ predicted ln(TG) + age + sex + BMI, where predicted ln(TG) was obtained from Stage 1. As a binary-outcome robustness check, a two-stage residual inclusion (2SRI/control-function) logistic model was additionally fit as: hypertension ~ ln(TG) + first-stage residual + age + sex + BMI, where residual is ln(TG) − predicted ln(TG). ORs and 95% CIs were obtained as exp(β) from the logistic regression coefficient for predicted ln(TG) (2SPS) or ln(TG) (2SRI). Residual *P* denotes the *P* value for the residual term in the 2SRI model.

BMI, body mass index; CI, confidence interval; GRS, genetic risk score; MR, Mendelian randomization; OR, odds ratio; SNP, single nucleotide polymorphism; TG, triglycerides.
